# Real world costs and cost-effectiveness of Rituximab for diffuse large B-cell lymphoma patients: a population-based analysis

**DOI:** 10.1186/1471-2407-14-586

**Published:** 2014-08-12

**Authors:** Sara Khor, Jaclyn Beca, Murray Krahn, David Hodgson, Linda Lee, Michael Crump, Karen E Bremner, Jin Luo, Muhammad Mamdani, Chaim M Bell, Carol Sawka, Scott Gavura, Terrence Sullivan, Maureen Trudeau, Stuart Peacock, Jeffrey S Hoch

**Affiliations:** Pharmacoeconomics Research Unit, Cancer Care Ontario, Toronto, Canada; Centre for Excellence in Economic Analysis Research, St Michael’s Hospital, Kragujevac, Canada; Canadian Centre for Applied Research in Cancer Control, Toronto, Canada; Department of Surgery, Surgical Outcomes Research Center, University of Washington, Seattle, WA USA; Toronto Health Economics and Technology Assessment Collaborative, Toronto, Canada; Clinical Decision Making and Health Care, Toronto General Hospital, Toronto, Canada; Institute of Health Policy, Management and Evaluation, University of Toronto, Toronto, Canada; Department of Radiation Oncology, Princess Margaret Hospital, Toronto, Canada; Department of Oncology, Niagara Health System, St Catharines, Canada; Division of Medical Oncology & Hematology, Princess Margaret Hospital, Toronto, Canada; Institute for Clinical Evaluative Sciences, Toronto, Canada; Department of Medicine, Mount Sinai Hospital, Toronto, Canada; Provincial Drug Reimbursement Programs, Cancer Care Ontario, Toronto, Canada; McGill University, Montreal, Canada; Sunnybrook Health Sciences Centre, Toronto, Canada; British Columbia Cancer Agency, Vancouver, Canada; University of British Columbia, Vancouver, Canada

## Abstract

**Background:**

Current treatment of diffuse-large-B-cell lymphoma (DLBCL) includes rituximab, an expensive drug, combined with cyclophosphamide, doxorubicin, vincristine, and prednisone (CHOP) chemotherapy. Economic models have predicted rituximab plus CHOP (RCHOP) to be a cost-effective alternative to CHOP alone as first-line treatment of DLBCL, but it remains unclear what its real-world costs and cost-effectiveness are in routine clinical practice.

**Methods:**

We performed a population-based retrospective cohort study from 1997 to 2007, using linked administrative databases in Ontario, Canada, to evaluate the costs and cost-effectiveness of RCHOP compared to CHOP alone. A historical control cohort (n = 1,099) with DLBCL who received CHOP before rituximab approval was hard-matched on age and treatment intensity and then propensity-score matched on sex, comorbidity, and histology to 1,099 RCHOP patients. All costs and outcomes were adjusted for censoring using the inverse probability weighting method. The main outcome measure was incremental cost per life-year gained (LYG).

**Results:**

Rituximab was associated with a life expectancy increase of 3.2 months over 5 years at an additional cost of $16,298, corresponding to an incremental cost-effectiveness ratio of $61,984 (95% CI $34,087‒$135,890) per LYG. The probability of being cost-effective was 90% if the willingness-to-pay threshold was $100,000/LYG. The cost-effectiveness ratio was most favourable for patients less than 60 years old ($31,800/LYG) but increased to $80,600/LYG for patients 60–79 years old and $110,100/LYG for patients ≥80 years old. We found that post-market survival benefits of rituximab are similar to or lower than those reported in clinical trials, while the costs, incremental costs and cost-effectiveness ratios are higher than in published economic models and differ by age.

**Conclusions:**

Our results showed that the addition of rituximab to standard CHOP chemotherapy was associated with improvement in survival but at a higher cost, and was potentially cost-effective by standard thresholds for patients <60 years old. However, cost-effectiveness decreased significantly with age, suggesting that rituximab may be not as economically attractive in the very elderly on average. This has important clinical implications regarding age-related use and funding decisions on this drug.

**Electronic supplementary material:**

The online version of this article (doi:10.1186/1471-2407-14-586) contains supplementary material, which is available to authorized users.

## Background

Combination chemotherapy with cyclophosphamide, doxorubicin, vincristine, and prednisone (CHOP) is the standard care for diffuse large B cell lymphoma (DLBCL), an aggressive, common form of non-Hodgkin lymphoma. In the last decade, four randomized controlled trials (RCTs) and two small observational studies demonstrated that the addition of the humanized monoclonal antibody rituximab to this combination (RCHOP) significantly improved the overall survival of patients undergoing primary treatment, although very elderly patients (≥80 years) were underrepresented [1–7]. Our recent population-based study (n = 4,021) showed that RCHOP was associated with a significant increase in overall survival compared to CHOP in all ages, including ≥80 years, without evidence of any significant increase in serious toxicity detected [8].

However, the high cost of rituximab brings its cost-effectiveness into question. This is problematic because cost-effectiveness information is a critical complement of comparative effectiveness research for producing efficient care and promoting fairness; it supports clinicians’ professional commitment to fair distribution of finite resources and helps health care payers and plans ensure value for money [9, 10]. Economic models comparing RCHOP to CHOP have found RCHOP to be either a dominant strategy [11], or a cost-effective alternative to CHOP [12–15], but these models have relied on efficacy findings from RCTs and required assumptions regarding resource use since economic data were not prospectively collected. This is particularly relevant given the repeated demonstrations that patients who are eligible for RCTs are not representative of the wider population expected to use the treatment [16]. While these economic models may be useful in informing coverage decisions, they may not represent the true cost-effectiveness of rituximab in practice. There remains a lack of evidence needed by payers to assess the extent to which the innovation is medically beneficial and financially sustainable for typical patients in routine clinical settings.

We evaluated the real-world cost-effectiveness of rituximab in patients with newly diagnosed DLBCL, using routinely collected widely available data. Our objective was to provide an assessment of value for money and accountability for spending on rituximab for DLBCL in practice from a population-based health care system’s perspective using administrative data on real world patients.

## Methods

### Data sources

Our study received research ethics board approval from St. Michael’s Hospital and Sunnybrook and Women’s College Health Sciences Centre. All Ontario residents are covered for medically-necessary health care through a universal government-sponsored insurance plan [17]. This retrospective cohort study used linked data from several population-based administrative health-care databases (see Additional file 1: Table S1), and cancer specific databases (see Additional file 1: Table S2) in the province of Ontario, Canada. Permissions were received from the Institute for Clinical Evaluative Sciences, Cancer Care Ontario, and the Princess Margaret Hospital to use the data. All cost components included in this article were fully covered by the Ontario Ministry of Health and Long-Term Care during the study period. All intravenous cancer drugs were administered in cancer centres or hospitals.

### Study cohort

Rituximab was approved for public funding via the New Drug Funding Program (NDFP) for patients with DLBCL on three different dates: Jan 10, 2001 (for 60–80 years old), April 2, 2001 (for ≥80 years old), and July 1, 2004 (for all ages). A historical cohort design was used to compare the outcomes of patients receiving CHOP before rituximab approval (CHOP group) with the outcomes of patients receiving RCHOP after rituximab approval (RCHOP group). The last follow-up date was March 31st, 2009.

The RCHOP group included patients who received their first dose of rituximab as first-line treatment for DLBCL from the date of rituximab approval for each age group to December 31st, 2007. The control patients (i.e., CHOP group) received CHOP-based chemotherapy as first-line treatment from January 1, 1997 to the date of rituximab approval for each age group, and had no evidence of receiving rituximab. All patients were required to have an Ontario Cancer Registry (OCR) record of new DLBCL diagnosis within six months prior to and up to 30 days after their first RCHOP or CHOP treatment, and those with missing data on histological diagnosis, Ontario Health Insurance Plan (OHIP) number, or sex were excluded. Furthermore, patients with a previous diagnosis of HIV infection any time prior to their first DLBCL diagnosis or lymphoma more than a year prior to their first DLBCL diagnosis (defined as ICD-9 histology codes: 9590–9769) were excluded. Further details are reported elsewhere [8].

### Outcomes

The medical resources included in the cost analysis are listed in Table S1 (see Additional file 1: Table S1). Only direct medical costs were included, and all costs were converted to 2009 Canadian dollars. The total direct medical costs for each patient in each study arm were estimated as the sum of all cost categories. Analyses of total health care costs can be challenging because patient data are often censored due to the brief nature of the follow-up. Censoring arises because of the inability to follow all patients until the endpoint of interest (e.g. death). Without appropriately adjusting for censoring, severely biased estimates of the mean total costs can arise. We applied the Inverse Probability Weighting (IPW) nonparametric method to adjust for censoring in our cost data [18]. This method accounts for censoring by weighting uncensored costs by the inverse probability of inclusion. To do this, the study period is often partitioned and the total observed cost in each time interval is divided by the probability of not being censored at the beginning of the interval to arrive at the adjusted costs for each interval. Mean cost is then estimated by summing the totals across all intervals and then dividing the sum by the sample size. In our study, observation time was partitioned and interval boundaries were chosen to coincide with censoring times. Weights were constructed separately for each treatment group, and 3-year and 5-year costs were estimated.

Overall survival was estimated using the Kaplan-Meier method for each cohort. Survival was defined as the time from diagnosis to date of death from any cause or the end of the study timeframe. To estimate mean survival time, the survival data were partitioned the same way as the cost data. Mean 3-year and 5-year survival times were determined using the same IPW methodology. Discounting was applied at 3% per year to both life years and costs.

The impacts of patient age and study timeframe (3 vs. 5 years) on costs and survival were examined. These restricted time points were chosen such that the standard errors of the survival estimates at these time points in each group were within reasonable limits (e.g. no larger than 5-10%) [19].

### Statistical analyses

To determine the adjusted association of rituximab with the primary outcomes, the treatment groups were first hard-matched by age group and disease severity at date of DLBCL diagnosis. Neither stage of disease nor International Prognostic Index (IPI) was available in the OCR for the years of our study. We used treatment intensity as a proxy for severity of the disease: “low” for those who received 3–4 cycles of chemotherapy followed by radiation within 60 days; “high” for those who received ≥ 4 cycles of chemotherapy with or without radiation, and “unclassifiable” if two or fewer cycles were administered or if an individual received three or four cycles without radiation [8]. Propensity scores were then estimated for each group and subjects were matched on the estimated propensity to receive RCHOP versus CHOP [20]. Baseline characteristics including sex, income quintile by postal code of residence at date of diagnosis, Adjusted Clinical Group (ACG) scores within three years prior to diagnosis, and primary histological diagnosis code were entered as independent variables in a multivariate logistic regression model. RCHOP and CHOP patients were subsequently matched (1:1) on propensity scores, without replacement. Nearest neighbour matching using calipers of width 0.2 standard deviations of the logit of the propensity score was used [21]. All unmatched patients were removed from further analysis. Standardized differences were assessed for balance in the baseline characteristics of the treatment groups after propensity-score matching [20]. A standardized difference of less than 10% in a covariate was considered to represent good balance between treatment groups [22]. P-values were calculated using the Wilcoxon signed rank test for continuous variables and McNemar’s test for binary variables.

The incremental cost-effectiveness ratios (ICERs) were estimated by dividing the mean additional total costs by the additional mean life-years gained associated with rituximab. The 95% confidence intervals (CIs) for the ICERs were estimated using a non-parametric bootstrapping method with 1,000 replicates. Each bootstrap iteration included both the cost and survival of the matched pair. The results for CHOP compared to RCHOP were presented as a scatter plot on the cost-effectiveness plane and as cost-effectiveness acceptability curves.

## Results

The study consisted of 4,021 patients with DLBCL, of whom 2,825 were in the RCHOP group and 1,196 were in the CHOP group (Table 1). The differences between patient groups were significant (absolute standardized difference >10%) for three of the six baseline characteristics, suggesting that treatment status was confounded by factors prognostic of DLBCL mortality. Patients who received RCHOP were older, and had more comorbidity and different histology. We matched 1,099 patients in the CHOP group (92%) to 1,099 patients who received RCHOP. There were no significant differences in measured characteristics between treatment groups after matching.

**Table 1:** **Baseline characteristics of CHOP and RCHOP patients before and after age, treatment intensity and propensity score matching**

		Before matching				After matching			
Characteristics		CHOP	RCHOP	Std. diff	***P value***	CHOP	RCHOP	Std. diff	***P value***
		N = 1,196	N =2,825			N = 1,099	N = 1,099		
*Age at diagnosis*									
	Mean ± SD	56 · 7 ± 16	65 · 5 ± 14	0 · 62	<·001	57 · 5 ± 16	58 · 6 ± 15	0 · 07	0 · 12
*Age group*									
	0-19	1%	<1%	0 · 09	<·001	<1%	<1%	0 · 00	1 · 00
	20-59	56%	25%	0 · 67		53%	53%	0 · 00	
	60-69	19%	30%	0 · 25		20%	20%	0 · 00	
	70-79	20%	33%	0 · 30		21%	21%	0 · 00	
	80+	5%	12%	0 · 22		6%	6%	0 · 00	
*Female*		47%	48%	0 · 01	0 · 74	47%	48%	0 · 00	0 · 93
*ACG group*									
	0	<1%	<1%	0 · 09	<·001	<1%	<1%	0 · 00	0 · 96
	1-3	7%	5%	0 · 10		7%	7%	0 · 02	
	4-6	24%	17%	0 · 18		23%	23%	0 · 00	
	7-9	28%	31%	0 · 05		29%	31%	0 · 03	
	10 +	40%	47%	0 · 16		41%	40%	0 · 02	
*Income quintile*									
	1	16%	17%	0 · 02	0 · 18	16%	15%	0 · 04	0 · 62
	2	20%	21%	0 · 02		19%	20%	0 · 02	
	3	20%	19%	0 · 02		21%	21%	0	
	4	24%	21%	0 · 08		23%	24%	0	
	5	20%	22%	0 · 06		20%	20%	0 · 01	
	Missing	<1%	<1%	0 · 02		<1%	<1%	0 · 07	
*Severity of disease*									
	Low	32%	30%	0 · 04	0 · 04	31%	31%	0 · 00	>0 · 99
	High	54%	58%	0 · 08		56%	56%	0 · 00	
	Unclassifiable	15%	12%	0 · 07		13%	13%	0 · 00	
*Histology code*									
	9590	16%	20%	0 · 11	<·001	16%	16%	0 · 01	0 · 92
	9591	3%	3%	0 · 02		3%	2%	0 · 03	
	9640	80%	69%	0 · 25		79%	80%	0 · 02	
	9680	2%	9%	0 · 29		2%	2%	0 · 00	
*Median Follow-up time (years)*		9 · 7	3 · 7			9 · 7	3 · 5		

*Std Diff*, standardized difference; *ACG*, adjusted clinical group; Severity of disease was estimated using treatment intensity; Histology codes based on International Classification of Disease diagnosis codes.

### Mean discounted survival

**Figure 1:**
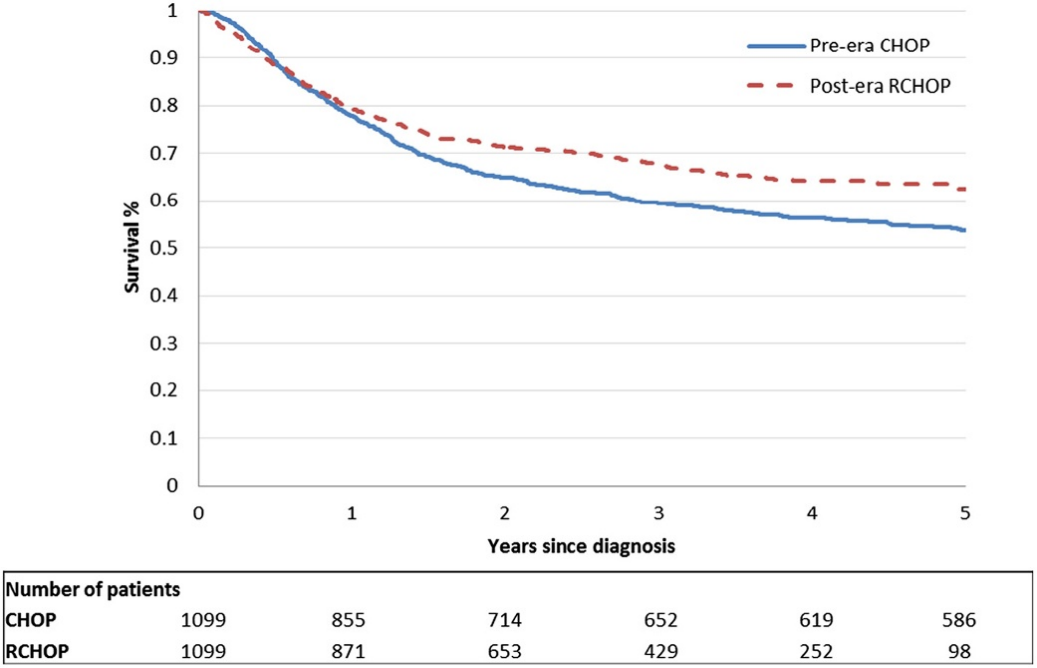
**Kaplan-Meier Survival functions for Pre-era CHOP and Post-era RCHOP patients.**

Figure 1 illustrates the overall survival functions and the number at risk by year for the two groups. The 3-year and 5-year mean survival of DLBCL patients treated with RCHOP were 2.28 and 3.44 years, respectively, compared with 2.16 and 3.18 years in the CHOP group (Table 2). RCHOP was associated with a mean absolute survival gain of approximately 1.3 months (95% CI 0.7-2.3) at three years and 3.2 months (95% CI 1.6-4.7) at five years. Age was associated with reductions in survival in both treatment arms in the 3- and 5-year time frames.

**Table 2:** **3-year and 5-year cost-effectiveness results by patient age at diagnosis**

Time frame		Age group	RCHOP	CHOP	Incremental (95% CI)	ICER (95% CI) ($/LYG)
**3 year**	*All ages*		n = 1,099	n = 1,099		134,136 (71,368 - 398,400)
		Mean costs	$76,815	$61,394	$15,421 (10,945 - 20,469)	
		Mean life-years	2 28	2 16	0 11 (0 06-0 19)	
	*<60*		n = 586	n = 586		73,037 (19,839 - 326,574)
		Mean costs	$72,121	$61,860	$10,261 (3,680 - 16,464)	
		Mean life-years	2 51	2 37	0 14 (0 04-0 23)	
	*60-79*		n = 450	n = 450		302,881 (90,094 - dominated)
		Mean costs	$82,149	$62,328	$19,822 (13,780 - 26,693)	
		Mean life-years	2 04	1 98	0 07 (−0 07 - 0 20)	
	*≥80*		n = 63	n = 63		128,766 (40,809 - dominated)
		Mean costs	$80,456	$50,381	$30,075 (14,048 - 49,693)	
		Mean life-years	1 78	1 55	0 23 (−0 11 - 0 61)	
**5 year**	*All ages*		n = 1,099	n = 1,099		61,984 (34,087 - 135,890)
		Mean costs	$85,293	$68,995	$16,298 (10,829 - 22,044)	
		Mean life-years	3 44	3 18	0 26 (0 13 - 0 39)	
	*<60*		n = 586	n = 586		31,789 (6,195 - 160,587)
		Mean costs	$77,453	$68,281	$9,172 (1,765 - 16,252)	
		Mean life-years	3 88	3 60	0 29 (0 09 - 0 47)	
	*60-79*		n = 450	n = 450		80,601 (34,420 - dominated)
		Mean costs	$90,818	$72,006	$18,812 (12,124 - 26,301)	
		Mean life-years	3 02	2 79	0 23 (−0 02 - 0 45)	
	*≥80*		n = 63	n = 63		110,071 (33,478 - dominated)
		Mean costs	$94,131	$54,114	$40,017 (18,077,68,826)	
		Mean life-years	2 46	2 10	0 36 (−0 22 - 0 98)	

*CHOP*, cyclophosphamide, doxorubicin, vincristine, and prednisone; *RCHOP*, CHOP with rituximab; *ICER*, incremental cost-effectiveness ratio; *CI*, confidence interval.

All values discounted (at r = 3%) and censored adjusted (with Inverse Probability Weighting). All costs are in 2009 Canadian dollars.

### Mean discounted costs

**Figure 2:**
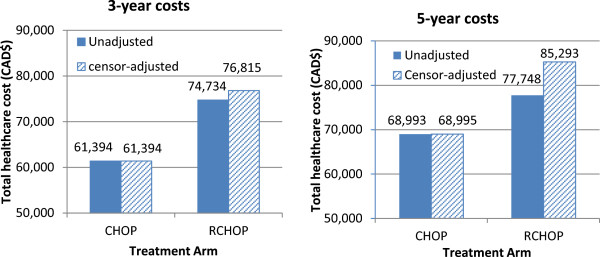
**The effect of adjusting for censoring in 3-year (left) and 5-year total costs (right).** Matched pre-era CHOP and post-era RCHOP patients; all ages; all values discounted.

The median follow-up time was 9.7 years for the CHOP cohort and only 3.5 years for the RCHOP cohort because rituximab was not approved for funding until 2001 to 2004. Therefore, the degrees of censoring in these two cohorts were different in the 3-year (0% vs. 30%) and 5-year (0.5% vs. 58%) time frames. Figure 2 illustrates the cost estimates before and after adjusting for censoring.

**Figure 3:**
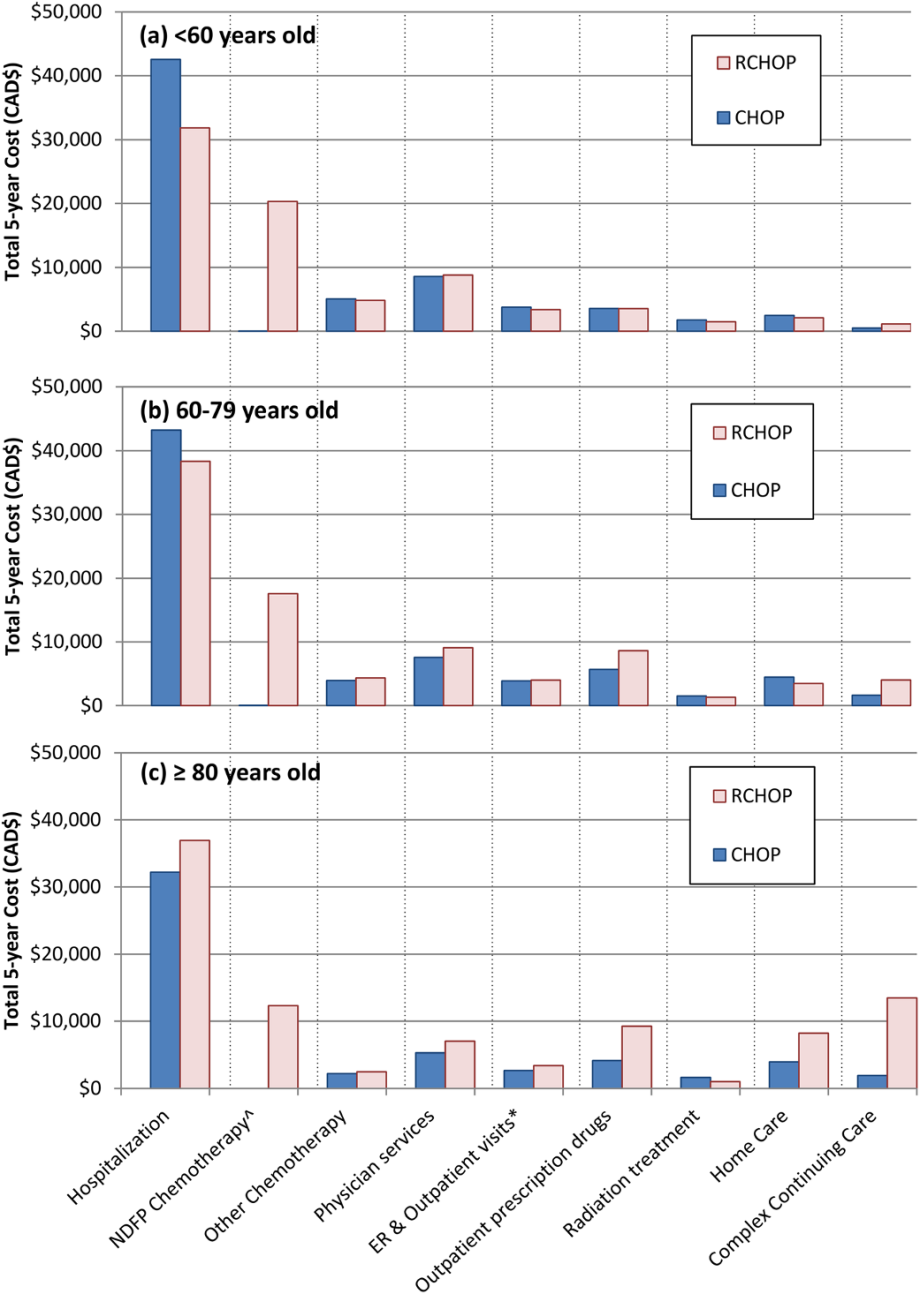
**Total cost by cost category for patients (a) <60, (b) 60–79, and (c) ≥80 years old.** All values discounted and censored adjusted. Blue bar represents CHOP patients; light pink bar represents R-CHOP patients.

The 3-year and 5-year mean censoring-adjusted costs of patients treated with RCHOP were $76,815 and $85,293, respectively, and $61,394 and $68,995 in those who received CHOP (Table 2). The incremental costs for RCHOP were $15,421 (95% CI 10,945-20,469) over 3 years and $16,298 (95% CI 10,829–22,044) over 5 years. Total costs increased with age for the RCHOP patients while they decreased with age for CHOP, corresponding to an increase in incremental costs with age (Table 2). Figure 3 shows the breakdown of costs by resource categories. The main cost driver, regardless of age or treatment group, was hospitalization. Young RCHOP patients had significantly lower hospitalization costs than CHOP patients, although the difference was not enough to offset the high costs of rituximab in the RCHOP group. Conversely, very elderly RCHOP patients had higher hospitalization costs than CHOP patients because hospitalization costs decreased with age among CHOP patients but increased with age among RCHOP patients. The cost of rituximab also decreased with age, and accounted for the major cost difference between the two treatment groups, except in the very elderly group, for whom the costs of home care and complex continuing care surpassed that of rituximab. Most of the costs were incurred during the first year following DLBCL diagnosis (Table 3).

**Table 3:** **Mean cost by year and patient age at diagnosis**

Mean cost (CAD$)		Year from diagnosis				
		1	2	3	4	5
***All Ages***						
	**CHOP**	45089	10890	5415	4052	3549
	**RCHOP**	63266	7733	5816	4343	4135
***<60 yrs old***						
	**CHOP**	44401	12249	5211	3633	2787
	**RCHOP**	60007	7031	5084	2825	2507
***60-79 yrs old***						
	**CHOP**	46488	9678	6161	4926	4752
	**RCHOP**	67364	8532	6254	4894	3774
***≥80 yrs old***						
	**CHOP**	41490	6902	1988	1699	2034
	**RCHOP**	64310	8465	7681	8142	5533

All values discounted (at r = 3%) and censored adjusted (with Inverse Probability Weighting). All costs are in 2009 Canadian dollars.

*CHOP*, cyclophosphamide, doxorubicin, vincristine, and prednisone; *RCHOP*, CHOP with rituximab.

### Cost-effectiveness

The ICER for all ages was $134,136/life-year gained (LYG) (95% CI 71,368 - 398,400) with a 3-year time horizon, and $61,984/LYG (95% CI: 34,087 - 135,890) with a 5-year time horizon (Table 2). This decrease over a longer time horizon reflects the concentration of costs in the first three years, while benefits extended into subsequent years. This held true for all age subgroups. However, the 5-year ICERs increased with age (Table 2), consistent with the increases in incremental costs with age.

**Figure 4:**
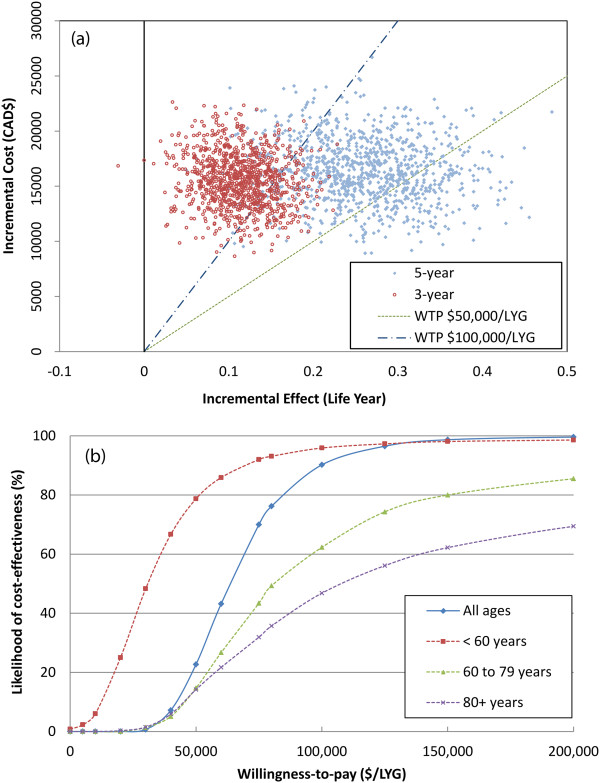
**Incremental cost-effectiveness ratio scatterplot and cost-effectiveness acceptability curves. (a)** Top - Scatterplot for incremental cost-effectiveness ratios (ICERs) for all ages based on bootstrapping. Each point represents the mean incremental cost and effectiveness of RCHOP compared to CHOP. A shift of distribution of ICERs from a 3-year to a 5-year timeframe is demonstrated. **(b)** Bottom - 5-year cost-effectiveness acceptability curves for different age groups. The curves represent the probability RCHOP is cost-effective compared with CHOP based on a willingness-to-pay threshold for the ICER.

Using the 5-year cost-effectiveness acceptability curves and assuming a willingness-to-pay threshold of $50,000/LYG, RCHOP was cost-effective in 23% of the bootstrap replications for all ages, 79% for the younger patients (<60 years), 15% for the elderly (60–79 years), and 14% for the very elderly (≥80 years) patients (Figure 4b). Assuming a willingness-to-pay threshold of $100,000/LYG, RCHOP was cost-effective in 90% of the replications for all ages, 96% for the younger patients, 62% for the elderly, and 47% for the very elderly.

## Discussion

Our overall results show that RCHOP for DLBCL was associated with a mean improvement in survival of approximately 3.2 months over a 5-year period but approximately $16,000 higher costs than standard CHOP chemotherapy, with an ICER of $62 K/LYG and a high probability of being cost-effective if the willingness-to-pay were at least $100 K for an extra year of life. However, cost-effectiveness decreased significantly with age, suggesting that the use of rituximab is not as economically attractive in the very elderly.

Our study had several strengths. First, our large population-based analysis included very elderly patients previously excluded from RCTs and young patients who were not captured in other databases such as Medicare. We also included a more comprehensive list of cost elements than previous cost-effectiveness studies [11, 14], which allowed us to analyse the cost components with respect to age and time up to five years. Costs from this study are not only relevant to countries with a universal single-payer healthcare system similar to Ontario’s, but also to systems with multiple payers in which these healthcare costs would be distributed among private insurers, government-sponsored insurance, and patient out-of-pocket costs. Second, this study used administrative datasets exclusively, rather than prediction models [11–14], to address the knowledge gap on the cost-effectiveness of rituximab for DLBCL in routine clinical practice. The results from this evaluation provide additional evidence needed to make or re-evaluate coverage decisions to ensure medical benefits, safety, and affordability of innovation. Third, we used a rigorous matching protocol to reduce bias [20]. Finally, we applied IPW to account for censoring in the cost and survival data. Although there are guidelines for the statistical analysis of censored cost data, few studies apply them [23].

There are several limitations to our study. First, the OCR for the study period did not contain stage data or full prognostic information (e.g. IPI score) for DLBCL patients, which are clinically useful predictors of survival outcomes that help guide treatment planning. We used ACG scores, a population-patient case-mix system, to estimate the burden of co-morbid illness, and used treatment intensity as a proxy for disease severity, but differences in treatment practices may lead to misclassification, although how this would bias the apparent incremental benefits and costs of rituximab is unclear. Since RCHOP is associated with improved survival compared to CHOP, it is possible that a CHOP patient who achieved the same number of cycles of therapy as his/her RCHOP match was actually healthier, and hence matching on treatment intensity could lead to estimates that might be biasing against rituximab. However, we expect this selection bias, if any, to be small. Second, outpatient prescription drug data were not available for most patients aged <65 years. However we expect minimal bias because we hard-matched the treatment groups by age. Third, we relied predominantly on Activity Level Reporting data to select our CHOP cohort, and therefore did not include patients from hospitals or clinics that did not submit data, potentially explaining the smaller size of the CHOP cohort before matching. However, our matched cohort was large, potentially improving its representativeness. Fourth, cost and survival benefits accumulated at a different rate in our study. While most costs were incurred in the first years after diagnosis, survival gains extend into later years. The ICER estimate is very sensitive to survival benefits, and it is possible that rituximab would be more favourable if the follow-up time was to extend beyond 5 years. Our approach measures, at best, a 5-year estimate. Finally, we did not use a contemporaneous cohort design due to rapid rituximab uptake post-approval. In fact, only a small subset (5-6%) of patients did not receive rituximab after 2005. These patients could be sicker, weaker, or have other health conditions, and using them as contemporary comparators could introduce unnecessary bias. With a historical cohort design, however, temporal improvement in patient management and differential censoring are challenges. To account for differential censoring, we limited our study time period and applied IPW to each treatment group separately. It is possible that recent widespread efforts in Ontario to shift end-of-life care from acute care settings to home care and community care centres [24], and to shift complex continuing care from a lighter care residential model to active rehabilitation of more medically complex patients partially explain the higher home care and continuing care costs we detected among our very elderly RCHOP patients [25], but these trends were not evident in the other age groups. Nonetheless, the costs we reported were the actual costs observed and we feel that our results represent valid estimates of the cost of care of RCHOP patients in the context of contemporary management for the period observed.

Compared to other studies that used same time horizons, our survival benefit from rituximab among young patients is lower than an Italian model (1.6 vs. 2.2 months at 3 years) [11], but it only included patients with good prognosis [3]. Our 5-year overall survival gain for elderly patients was similar to a study in British Columbia (BC) (0.2 vs 0.4 year) [15], but much lower than the 1.04 years reported in a US study that extrapolated survival data from the European phase III GELA trial [14]. Different modelling assumptions and extrapolation of trial data can generate a substantial variability in outcomes, highlighting the importance of validating findings with follow-up comparative effectiveness research such as this study.

While rituximab extended survival in all age groups, we found that its major impact on healthcare resource use was the reduction in hospitalization among patients <80 years old, especially for the youngest patients (<60 years). For the very elderly (≥80 years), however, RCHOP did not reduce hospitalization, while costs of other non-cancer resources significantly increased with age among RCHOP patients more than among CHOP patients, resulting in a high incremental cost for this age group. This is consistent with a recent Medicare study that reported more expensive non-chemotherapy-related and non-cancer related care among elderly rituximab patients as a result of longer survival [26]. In our elderly rituximab patients, some of the additional costs were offset by the reduction in hospitalization, partially explaining our lower incremental cost than the Medicare study (4-year: $20 K vs. Medicare $28 K) (all values converted to 2009 Canadian dollars and rounded). Also that study only included patients >65 years old. In contrast to the Medicare study, our very elderly patients experienced an even more significant increase in non-chemotherapy and non-cancer costs, resulting in our higher incremental costs (4-year $37 K vs. $25 K), and suggesting rituximab is not cost-effective by standard thresholds (Medicare ICER: $60 K/LYG vs. our ICER: $114 K/LYG). This may be related to the fact that very elderly patients who received RCHOP had greater survival benefit than other age groups, and continued to incur more cost-intensive medical costs due to age and other conditions.

We found that real-world costs, incremental costs and cost-effectiveness ratios are higher than in published economic models and differ by age [11, 14, 15]. For example, we did not observe lower costs in rescue therapy that could offset the high costs of rituximab to make it a cost-saving intervention for young patients, as projected by an Italian model [11], or lower costs in palliative care for the elderly patients that could significantly reduce incremental cost, as described by the US model [14]. These models, however, excluded key drivers of total and incremental costs such as the costs of hospitalization and prescription drugs. Compared to a British Columbia microsimulation, our 5-year incremental cost was comparable ($9 K vs. $10 K) for young patients [15], but significantly higher for elderly patients ($19 K vs. $8 K). That study projected a reverse trend of incremental costs and ICER with age ($51 K/LYG for young patients and $21 K/LYG for the elderly) than what we observed, but its cost estimates were based on aggregated and literature-based data, and it did not observe a relationship between non-chemotherapy cost components and age as did our observational study. Variations in the ICERs found in these economic analyses are driven by different model assumptions.

Cost-effectiveness results are sensitive to study timeframe. Since most costs were incurred within the first years following DLBCL diagnosis, a longer study horizon resulted in a more economically attractive assessment because benefits extend into subsequent years. In fact, a follow-up study on the GELA trial showed that the survival benefits of the addition of rituximab to CHOP persisted over a 10-year follow-up [27]. Our study’s goal was to highlight the usefulness of providing cost-effectiveness information alongside comparative effectiveness data that reflect routine clinical practice on representative patients, so we did not extrapolate beyond our data. Follow-up studies could examine the cost-effectiveness of rituximab over a longer time horizon and compare against findings in published models that used standardized methods for life-time projections of survival benefit and costs.

## Conclusions

While trial data and predictive modelling remain the gold standards for estimating clinical efficacy and costs in economic evaluations, decision-makers are increasingly seeking real-world evidence. Our real-world cost-effectiveness analysis demonstrates that post-market evaluations that reflect actual practice can produce results that differ from trials or prediction models, but results are sensitive to patients’ age and study timeframe. This type of post-market analysis can help calibrate policies (e.g., to re-evaluate decisions post-approval) and support healthcare payers’ mandate for accountability and sustainability, and they should become a more routine part of drug listing appraisals, contributing to a life cycle approach to drug evaluation. Our study also highlights the impact of appropriate methods to adjust for incomplete cost data and choice of timeframe on real-world cost-effectiveness results. These findings have important implications for establishing “coverage with evidence development” or “only in research” funding arrangements.

## Electronic supplementary material

Additional file 1: **This file contains two additional tables for the manuscript.** **Table S1.** Data sources and methods used for costing health-related resources. **Table S2.** Ontario Cancer Databases & Registered Persons Database. (PDF 124 KB)

## Abbreviations

DLBCL: Diffuse-Large-B Cell Lymphoma
CHOP: Cyclophosphamide, doxorubicin, vincristine, and prednisone
RCHOP: Rituximab plus CHOP
LYG: Life-year gained
RCT: Randomized controlled trials
NDFP: New Drug Funding Program
OCR: Ontario Cancer Registry
OHIP: Ontario Health Insurance Plan
IPW: Inverse probability weighting
ACG: Adjusted Clinical Group score
ICER: Incremental cost-effectiveness ratio
CI: Confidence interval.
