# Supplementary material for: Real world costs and cost-effectiveness of Rituximab for diffuse large B-cell lymphoma patients: a population-based analysis
Source: BMC Cancer. 2014 Aug 12;14:586. doi: 10.1186/1471-2407-14-586 (PMC4148552; doi:10.1186/1471-2407-14-586)
Supplement: Supplementary file 1 — Additional file 1: This file contains two additional tables for the manuscript. Table S1. Data sources and methods used for costing health-related resources. Table S2. Ontario Cancer Databases & Registered Persons Database. (PDF 124 KB) [file 12885_2013_4781_MOESM1_ESM.pdf]

Additional Material

eTable 1: Data sources and methods used for costing health-related resources

Category	Source for resource use	Source for costs
**Hospitalization**	CIHI Discharge Abstract database[1]	Resource Intensity Weight (RIW) methodology[2] using CIHI Discharge Abstract database
**Chemotherapy**	NDFP database[3]<br><br>Other outpatient chemotherapies: Pre-2003 OHIP database[4] (codes G281, G381, G339, G345 or G359);<br>Post-2003 NACRS database (code for “main problem” of Z51·1)	Cost per case from NDFP database<br><br>Post-2003 average cost per visit: RIW methodology using NACRS database[5]. Average then applied to all chemo visits pre-2003.
**Physician Services***	OHIP database	OHIP billing claims
**Emergency Room visits / Same Day Surgery or procedure**	Pre-2002 ER visits: OHIP database<br>Pre-2002 Same Day Surgery: CIHI Discharge Abstract database<br>Post-2002 ER and Same Day Surgery: NACRS database	Post-2002 average cost per visit: RIW methodology using NACRS database. Average then applied to all visits pre-2002.
**Prescription drugs****	Ontario Drug Benefit Plan (ODB)[6]	ODB prescription records
**Radiation treatment**	NHPIP codes and number of minutes for the procedure from the Activity Level Reporting database	Cost per visit: Estimated unit cost/minute from Earle et al.’s [7] inflated to 2009 dollar ($11·92) multiplied by the NHPIP unit for each patient
**Complex Continuing Care*****	Mean weighted-days: Continuing Care Reporting System (case-mix index multiplied by duration between events)	Cost per weighed-day from Hospital e-scorecard[8] inflated to 2009 dollar ($459·10)
**Home care services******	Ontario Home Care Administrative System & Home Care Database	Community Care Access Centre[9] database

* Physician Services include inpatient and outpatient laboratory / imaging services
** ODB covers prescription drug utilization of elderly and low income patients
***Complex Continuing Care includes long term medical care, geriatric assessment, rehabilitation, psychogeriatric, palliative and respite care
****Home care services include visiting professionals, personal, homemaking and community support
CIHI = The Canadian Institute for Health Information; NDFP = New Drug Funding Program; OHIP = Ontario Health Insurance Plan; NACRS = National Ambulatory Care Reporting Service; NHPIP = National Health Productivity Improvement Program

eTable 2: Ontario Cancer Databases & Registered Persons Database

Database	Description
**Ontario Cancer Registry (OCR)[10-12]**	Contains all cancer diagnoses in Ontario
**New Drug Funding Program database (NDFP)[3]**	Describes the use of intravenous cancer drugs
**Activity Level Reporting System (ALR)**	Describes chemotherapy and radiation therapy administered in 41 cancer centres and related general hospitals, capturing approximately 60% of the chemotherapy and 100% of the radiation therapy administered in Ontario
**Registered Persons Database (RPDB)**	Describes patients’ demographics and vital statuses

Web References
1. Juurlink D, Preya C, Croxford R, Chong A: **Canadian Institute for Health Information Discharge Abstract Database: a validation study**. In: *ICES Investigative Report.* Toronto, ON: Institute for Clinical Evaluative Sciences; 2006.
2. **Canadian MIS Database-Hospital Financial Performance Indicators, 1999-2000 to 2008-2009: Methodological Notes**. In.: Canadian Institute for Health Information; 2010: 1-34.
3. Evans WK, Nefsky M, Pater J, Browman G, Cowan DH: **Cancer Care Ontario's New Drug Funding Program: controlled introduction of expensive anticancer drugs**. *Chron Dis Can* 2002, **23**(4):152-156.
4. Lee R, Hutnik CML: **Projected cost comparison of selective laser trabeculoplasty versus glaucoma medication in the Ontario Health Insurance Plan**. *Can J Opthalmol-J Can Opthalmol* 2006, **41**(4):449-456.
5. Jacobs P, Yim R: **Using Canadian administrative databases to derive economic data for health technology assessments**. In. Ottawa: Canadian Agency for Drugs and Technologies in Health; 2009.
6. **Ontario Drug Benefit : The Program** [http://www.health.gov.on.ca/english/public/pub/drugs/odb.html]
7. Earle C, Coyle D, Smith A, Agboola O, Evans WK: **The cost of radiotherapy at an Ontario regional cancer centre: a re-evaluation**. *Critical Reviews in Oncology Hematology* 1999, **32**(2):87-93.
8. **Hospital e-Scorecard Report 2008: Complex Continuing Care: Financial Performance and Condition Technical Summary**. In.: Ontario Ministry of Health and Long-Term Care; 2008.
9. **CCAC Comparative Reports 2005/2006 YE Final**. In.: Ontario Ministry of Health and Long-Term Care; 2006.
10. Brenner DR, Tammemaegi MC, Bull SB, Pinnaduwaje D, Andrulis IL: **Using cancer registry data: agreement in cause-of-death data between the Ontario Cancer Registry and a longitudinal study of breast cancer patients**. *Chron Dis Can* 2009, **30**(1):16-19.
11. Cuffe S, Booth CM, Peng YW, Darling GE, Li G, Kong WD, Mackillop WJ, Shepherd FA: **Adjuvant Chemotherapy for Non-Small-Cell Lung Cancer in the Elderly: A Population-Based Study in Ontario, Canada**. *J Clin Oncol* 2012, **30**(15):1813-1821.
12. Hall S, Schulze K, Groome P, Mackillop W, Holowaty E: **Using cancer registry data for survival studies: the example of the Ontario Cancer Registry**. *J Clin Epidemiol* 2006, **59**(1):67-76.
